# Advanced lung cancer inflammation index is associated with long-term cardiovascular death in hypertensive patients: national health and nutrition examination study, 1999–2018

**DOI:** 10.3389/fphys.2023.1074672

**Published:** 2023-05-03

**Authors:** Jiabin Tu, Bo Wu, Jiaming Xiu, Jiayi Deng, Shuqiong Lin, Jin Lu, Yanfang Yan, Pei Yu, Jinlong Zhu, Kaihong Chen, Shan Ding, Liling Chen

**Affiliations:** ^1^ Longyan First Affiliated Hospital of Fujian Medical University, Longyan, China; ^2^ Zhangzhou Affiliated Hospital to Fujian Medical University, Zhangzhou, China; ^3^ Zhongshan Hospital (Xiamen), Fudan University, Xiamen, China

**Keywords:** hypertension, inflammation, cardiovascular death, advanced lung cancer inflammation index, NHANES

## Abstract

**Background:** Hypertension is one of the main causes of cardiovascular death. Inflammation was considered influential factors of cardiovascular (CVD) death in patients with hypertension. Advanced lung cancer inflammation index (ALI) is an index to assess inflammation, few studies have investigated the relationship between advanced lung cancer inflammation index and cardiovascular death in hypertensive patients.

**Objective:** The aim of this study was to investigate the association between advanced lung cancer inflammation index and long-term cardiovascular death in hypertensive patients.

**Method:** Data from the National Health and Nutrition Examination Survey (NHANES) 1999–2018 with mortality follow-up through 31 December 2019 were analyzed. Advanced lung cancer inflammation index was calculated as BMI (kg/㎡) × serum albumin level (g/dL)/neutrophil to lymphocyte ratio (NLR). A total of 20,517 participants were evaluated. Patients were divided into three groups based on tertiles of advanced lung cancer inflammation index as follows: T1 (*n* = 6,839), T2 (*n* = 6,839), and T3 (*n* = 6,839) groups. The relationship between advanced lung cancer inflammation index and long-term cardiovascular death was assessed by survival curves and Cox regression analysis based on the NHANES recommended weights.

**Results:** The median advanced lung cancer inflammation index value in this study was 61.9 [44.4, 84.6]. After full adjustment, the T2 group (hazard ratio [HR]: 0.59, 95% confidence interval [CI]: 0.50–0.69; *p* < 0.001) and T3 group (HR: 0.48, 95% CI: 0.39–0.58; *p* < 0.001) were found to have a significantly lower risk of cardiovascular death compared to the T1 group.

**Conclusion:** High levels of advanced lung cancer inflammation index were associated with reduced risk of cardiovascular death in hypertensive patients.

## Introduction

Hypertension is a chronic disease that can be effectively intervened, and it is one of the primary causes of death from cardiovascular disease (CVD) (Shin et al., 2019). In fact, approximately half of all CVD deaths were related to hypertension (Kearney et al., 2005; Lim et al., 2012). Despite significant advancements in hypertension treatment and management, its impact on CVD mortality continues to rise worldwide. (Mills et al., 2016; Das, 2017).

Past research has revealed that inflammation plays a crucial role in the onset and progression of hypertension. Chronic inflammation can significantly elevate the risk of death from CVD. (Virdis et al., 2014; Boos et al., 2021). The majority of present-day studies that employ inflammatory markers to evaluate the prognosis of hypertension only utilize individual inflammatory markers (Engström et al., 2006; Cortez et al., 2016; Sun et al., 2017). Nevertheless, inflammation can result in decreased albumin and weight loss (Lennie, 1998; Sheinenzon et al., 2021). and relying on a single inflammatory marker may not provide sufficient precision to evaluate the prognosis of patients with hypertension.

The advanced lung cancer inflammation index, an index that combines body weight, albumin and neutrophil to lymphocyte ratio (NLR), was originally used to assess systemic inflammation levels in cancer patients (Jafri et al., 2013). In previous studies, ALI showed good efficacy in assessing inflammatory status in coronary artery disease and heart failure patients, and was associated with prognosis in these patients (Maeda et al., 2020; Fan et al., 2021). Given that hypertension was believed to have a connection with inflammation, we utilized the ALI to evaluate the inflammatory status among patients with hypertension, and investigated its correlation with death from CVD in hypertensive patients.

The purpose of this study was to investigate the relationship between ALI and the risk of CVD death in patients with hypertension and to provide some reference for the treatment and management of hypertensive patients.

## Materials and methods

### Study population

NHANES is a nationally representative cross-sectional survey recursively conducted in the United States by the National Center for Health Statistics. NHANES is based on a stratified multistage random sampling design. A retrospective analysis was performed using publicly available data from NHANES from 1999 to 2018.

**FIGURE 1:**
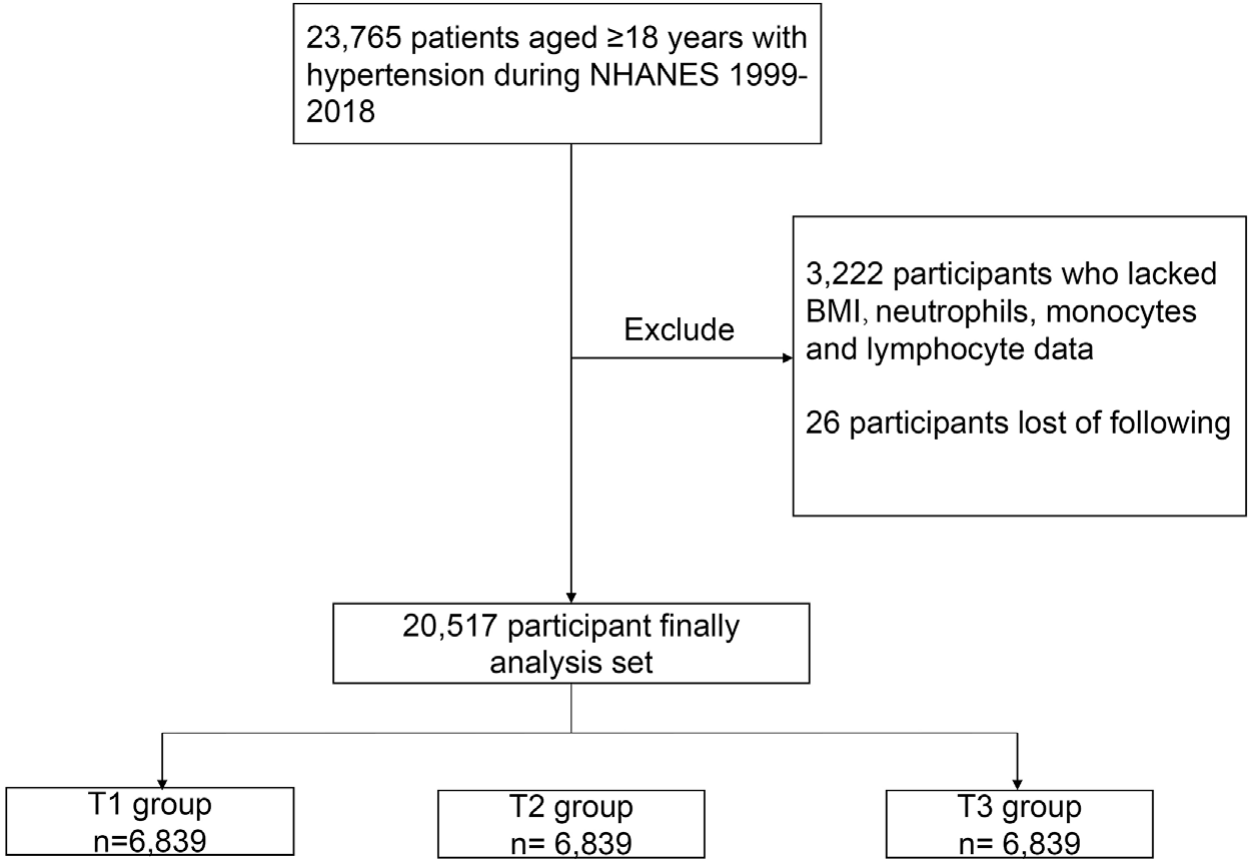
Flowchart of the study design.

In NHANES 1999–2018, our analysis was limited to 23,765 participants aged 18 years and older with hypertension. Hypertension was defined as an affirmative answer by participants to the question “Have you ever been told by a doctor or other health professional that you have hypertension, also called high blood pressure?” In addition, participants with systolic blood pressure (SBP) ≥140 mmHg or/and diastolic blood pressure (DBP) ≥90 mmHg were defined as having hypertension. If the participants had their blood pressure measured more than one time, their average blood pressure was used to determine whether the patient had hypertension (Unger et al., 2020). In addition, participants who were receiving antihypertensive medications are also considered to have hypertension. Of these participants, 3,222 people who lacked body mass index (BMI), albumin, neutrophil, and lymphocyte data were excluded. In addition, 26 participants who were lost follow-up were excluded. Ultimately, a total of 20,517 participants were included in this cohort study (Figure 1).

### Calculation of ALI

ALI was calculated as BMI (kg/m^2^) × serum albumin level (g/dL)/neutrophil-to-lymphocyte ratio (NLR). Patients were divided into three groups based on the tertiles of ALI: T1 group (≤50.0), T2 group (>50.0 and ≤77.0), and T3 group (>77.0).

### Primary outcome

The primary outcome was CVD death. Cause of death was categorized using the International Classification of Diseases 10th edition (ICD-10). Cardiovascular mortality was categorized using ICD-10 codes I00–I078. For participants in NHANES 1999–2018, mortality follow-up data was available through 31 December2019.

### Definitions of variables of interest

Age, sex, race, smoking status and drink status were self-reported by participants. Participants who were currently taking calcium channel blockers (CCB), diuretics, beta blockers, and angiotensin converting enzyme inhibitors (ACEI)/angiotensin II receptor blockers (ARB) were considered to be taking antihypertensive drugs. Laboratory measurements, such as creatinine (Cr), triglyceride (TG), total cholesterol (TC), blood glucose (Glu), albumin, neutrophil counts, and lymphocyte counts, were collected using automated hematological analysis equipment. Detailed procedures for obtaining laboratory measurements were provided in a document on the website of the National Center for Health Statistics. The Healthy Eating Index (HEI-2015) was calculated based on the patient’s total nutrient intake on the first day. Diagnosis of comorbidities was based on an affirmative response to the question “Has a doctor or other health professional ever told you that you had chronic heart failure (CHF), chronic heart disease (CHD), diabetes mellitus (DM), stroke, or cancer?”

### Statistical analyses

We used the NHANES recommended weights to calculate the weights for specific groups. Continuous variables were expressed as the mean ± standard deviation. Variables that do not conform to the normal distribution are represented by the median (25th percentile, 75th percentile). Categorical variables were presented as counts (percentages). Baseline characteristics between the three groups were compared using an analysis of variance (ANOVA) for continuous variables and an χ2 test for categorical variables.

To evaluate the association between ALI and long-term CVD death, we used Kaplan-Meier and Cox regression analyses. Both estimates and probabilities were based on weights recommended by NHANES. Model 1 was a crude model unadjusted for potential confounders. Model 2 was adjusted for age and sex. Model 3 was fully adjusted for potential confounders. Furthermore, we explored the relationship between ALI and CVD mortality in different subgroups including age, sex, BMI, antihypertensive drug and DM. Restricted regression cubic splines were used to explore the potential non-linear relationship between ALI and CVD death in hypertensive patients. COX regression analysis was performed on the variables required for ALI calculation.

All data analyses were performed by using the Survey package in R software (version 4.0.4; R Foundation for Statistical Computing, Vienna, Austria). A two-sided *p*-value <0.05 indicated significance for all analyses.

## Results

### Participant characteristics

Among all 20,517 participants eligible for the study, the average age was 54.8 ± 0.2 years. The distribution of ALI is shown in Supplementary Figure S1. Approximately half (*n* = 10,379, 50.9%) were female. Patients were divided into three groups based on the tertiles of ALI: T1 group (*n* = 6,839), T2 group (*n* = 6,839), and T3 group (*n* = 6,839). The median ALI of the T2 [62.5 (IQR:56.4–69.1)] and T3 [97.8 (IQR:85.6–119.7)] groups was found to be higher than that of the T1 group [38.0 (IQR:29.9–44.4)]. Participants in the group with higher ALI were younger (T1 group: 61.1 ± 0.3 vs. T2 group: 55.8 ± 0.3 vs. T3 group: 53.5 ± 0.3 years) and had higher BMI (T1 group: 27.7 ± 0.1 vs. T2 group: 30.9 ± 0.1 vs. T3 group: 33.8 ± 0.1 kg/m^2^) and were more likely to be female (T1 group: 49.8% vs. T2 group: 49.6% vs. T3 group: 53.6%). In the group with higher ALI, participants have lower Cr (T1 group: 89.61 ± 0.99 vs. T2 group: 81.30 ± 0.43 vs. T3 group: 79.41 ± 0.39 μmol/L) and Healthy Eating Index–2015 (HEI-2015) (T1 group: 51.3 ± 0.2 vs. T2 group: 50.8 ± 0.3 vs. T3 group: 50.3 ± 0.3). With increased ALI, the proportion of smokers gradually deceased (T1 group: 54.3% vs. T2 group: 50.4% vs. T3 group: 46.7%) and was less likely to be combined with stroke (T1 group: 7.8% vs. T2 group: 5.2% vs. T3 group: 4.4%), CHD (T1 group: 10.2% vs. T2 group: 6.5% vs. T3 group: 5.3%), CHF (T1 group: 7.0% vs. T2 group: 4.2% vs. T3 group: 3.9%) and cancer (T1 group: 19.7% vs. T2 group: 13.4% vs. T3 group: 10.9%). There was no statistical difference in DM among the three groups. In addition, the group with higher ALI levels had lower rates of CCB (T1 group: 19.4% vs. T2 group: 16.9% vs. T3 group: 14.9%) and *β*-block use (T1 group: 26.8% vs. T2 group: 21.8% vs. T3 group: 20.8%). There was no statistical difference in the number of people using ACEI/ARB and diuretics. More data on the baseline characteristics of study population are detailed in Table 1.

**TABLE 1:** Baseline characteristics of the study population (weighted).

	Overall (n = 20,517)			T1 group (*n* = 6,839)	T2 group (*n* = 6,839)	T3 group (*n* = 6,839)	*p*-value
ALI, mean [IQR:25%–75%]	61.9 [44.4, 84.6]			38.0 [29.9, 44.4]	62.5 [56.4, 69.1]	97.8 [85.6, 119.7]	<0.001
Age, (years), mean (SE)	56.8 ± 0.2			61.1 ± 0.3	55.8 ± 0.3	53.5 ± 0.3	<0.001
Female, n (%)	10,379 (50.9)			3,188 (49.8)	3,448 (49.6)	3,43 (53.6)	<0.001
Race, n (%)							<0.001
Mexican American	2,990 (5.6)			880 (4.6)	1,170 (6.4)	940 (5.9)	
Non-Hispanic Black	5,021 (12.8)			1,042 (7.2)	1,374 (10.0)	2,605 (21.7)	
Non-Hispanic White	9,459 (71.1)			3,966 (79.2)	3,199 (72.4)	2,294 (61.2)	
Other Hispanic	1,515 (4.4)			457 (3.6)	546 (4.9)	512 (4.7)	
Other Race	1,532 (6.0)			494 (5.4)	550 (6.2)	488 (6.4)	
BMI, (kg/㎡), mean (SE)	30.8 ± 0.1			27.7 ± 0.1	30.9 ± 0.1	33.8 ± 0.1	<0.001
SBP, (mmHg), mean (SE)	134.3 ± 0.2			135.5 ± 0.4	133.6 ± 0.4	133.6 ± 0.3	<0.001
DBP, (mmHg), mean (SE)	74.2 ± 0.2			72.2 ± 0.3	74.6 ± 0.3	75.8 ± 0.3	<0.001
Neutrophil, (K/μL), mean (SE)	4.44 ± 0.02			5.36 ± 0.03	4.41 ± 0.03	3.51 ± 0.02	<0.001
Lymphocyte, (K/μL), mean (SE)	2.14 ± 0.02			1.64 ± 0.01	2.12 ± 0.01	2.69 ± 0.04	<0.001
Albumin, (g/dL), mean (SE)	4.23 ± 0.01			4.18 ± 0.01	4.25 ± 0.01	4.26 ± 0.01	<0.001
Cr, (μmol/L), mean (SE)	83.47 ± 0.42			89.61 ± 0.99	81.30 ± 0.43	79.41 ± 0.39	<0.001
TG, mmol/L, mean (SE)	1.92 (0.02)			1.77 (0.02)	1.99 (0.03)	2.02 (0.03)	<0.001
TC, mmol/L, mean (SE)	5.14 (0.014)			5.03 (0.021)	5.172 (0.020)	5.23 (0.02)	<0.001
Glu, mmol/L, mean (SE)	5.91 (0.02)			5.953 (0.03)	5.932 (0.04)	5.826 (0.03)	<0.001
HEI-2015, mean (SE)	50.8 ± 0.2			51.3 ± 0.2	50.8 ± 0.3	50.3 ± 0.3	0.004
Smoke status, n (%)	10,170 (50.5)			3,699 (54.3)	3,351 (50.4)	3,120 (46.7)	<0.001
Drink status, n (%)	11,275 (67.8)			3,683 (67.1)	3,812 (69.3)	3,780 (66.9)	0.082
DM, n (%)	5,966 (23.8)			1938 (23.8)	1973 (23.1)	2055 (24.7)	0.221
CHF, n (%)	1,276 (5.1)			584 (7.0)	371 (4.2)	321 (3.9)	<0.001
CHD, n (%)	1,608 (7.3)			721 (10.2)	522 (6.5)	365 (5.3)	<0.001
Stroke, n (%)	1,452 (5.8)			640 (7.8)	451 (5.2)	361 (4.4)	<0.001
Cancer, n (%)	2,805 (14.7)			1,307 (19.7)	827 (13.4)	671 (10.9)	<0.001
CCB, n (%)	4,196 (20.5)			1,516 (19.4)	1,408 (16.9)	1,272 (14.9)	<0.001
Beta blockers, n (%)	4,921 (24.0)			1899 (26.8)	1,578 (21.8)	1,444 (20.8)	<0.001
ACEI/ARB, n (%)	8,436 (41.1)			2,867 (41.1)	2,823 (39.3)	2,746 (39.2)	0.481
Diuretics, n (%)	6,028 (29.4)			2021 (28.5)	1970 (27.2)	2037 (27.9)	0.796
Antihypertensive drugs, n (%)	13,023 (63.5)			4,517 (64.5)	4,261 (59.5)	4,245 (59.8)	<0.001
CVD death, n (%)	1,453 (6.5)			739 (3.3)	424 (1.9)	290 (1.3)	<0.001

Note: T1 group (ALI ≤ 50.00), T2 group (ALI > 50.00, and ≤77.00), T3 group (ALI > 77.00).

The average number of drinks consumed per day over the past 12 months.

Abbreviations: BMI, body mass index; SBP, systolic blood pressure; DBP, diastolic blood pressure; ALB, albumin; Cr, creatinine; TG, triglyceride; TC, total cholesterol; HEI-2015, healthy Eating Index-2015; DM, diabetes mellitus; CHF, congestive heart failure; CHD, coronary heart disease; CCB, calcium channel blockers; ACEI, angiotensin converting enzyme inhibitors; ARB, Angiotensin II, receptor blockers.

### ALI and CVD mortality

**FIGURE 2:**
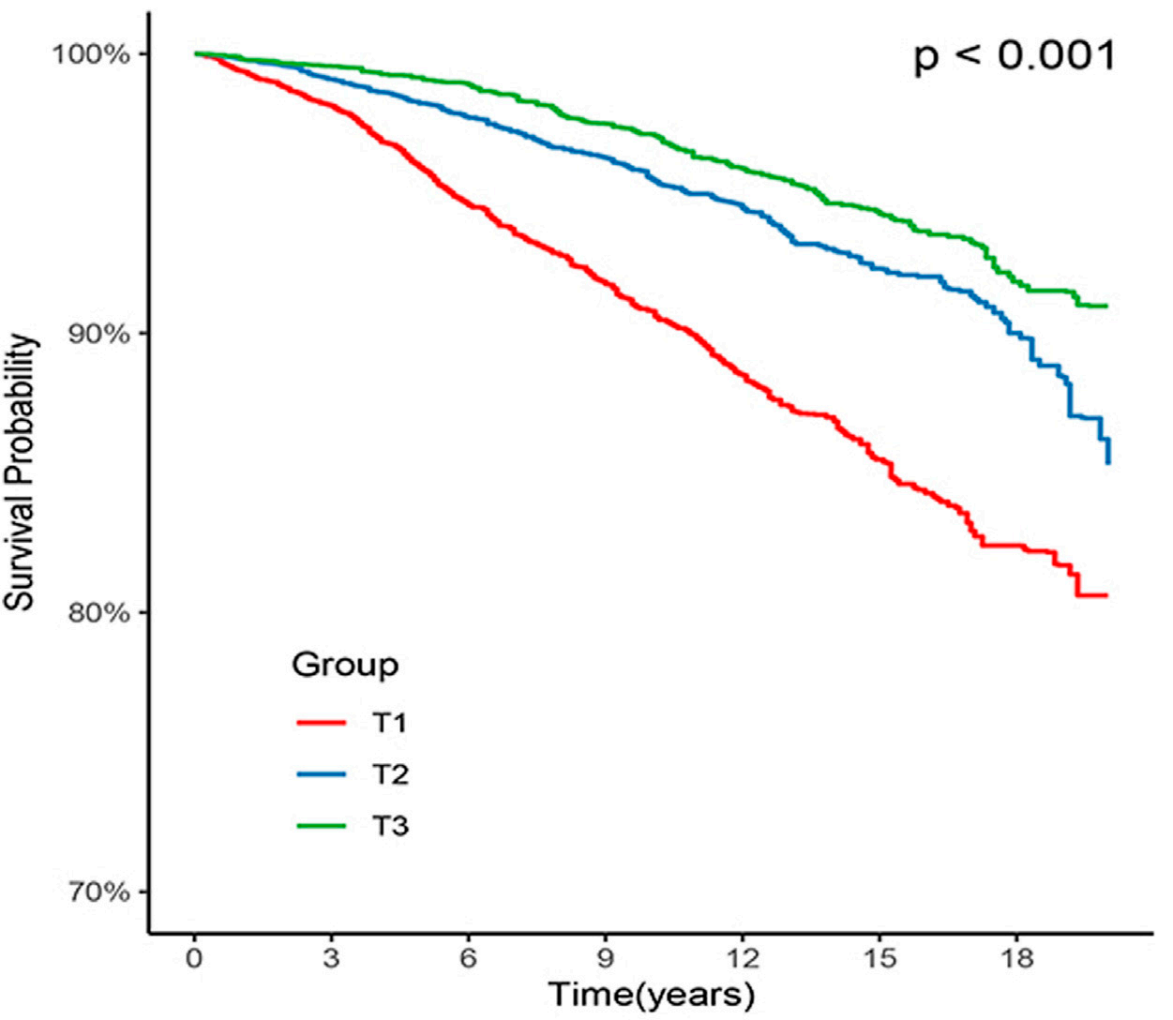
Kaplan-Meier survival estimates for long-term cardiovascular mortality (weighted).

Out of all the participants, 1,453 (6.5%) individuals died due to CVD, with 739 (3.3%) in the T1 group, 424 (1.9%) in the T2 group, and 290 (1.3%) in the T3 group. Kaplan-Meier survival analysis curves revealed that the groups with higher ALI had lower mortality rates from CVD (P-log rank <0.001, Figure 2). The results of univariate Cox proportional hazard analysis showed that the risk of death from CVD decreased by 16% for each 10 unit increase in ALI (95% confidence interval (CI): 0.81–0.87; *p* < 0.001). In comparison to the T1 group, both the T2 (hazard ratio (HR): 0.47, 95% CI: 0.41–0.53; *p* < 0.001) and T3 (HR: 0.32, 95% CI: 0.27–0.38; *p* < 0.001) groups had a lower risk of death from CVD. After adjusting for potential confounders including age, sex, ethnicity, smoking, drinking, Cr, TG, TC, Glu, CHF, CHD, DM, stroke, antihypertensive drugs, cancer, HEI-2015, DBP, and SBP, the risk of CVD death decreased by 11% (95% CI: 0.87–0.92; *p* < 0.001) for each 10 unit increase in ALI. The T2 (HR: 0.59, 95% CI: 0.50–0.69; *p* < 0.001) and T3 (HR: 0.48, 95% CI: 0.39–0.58; *p* < 0.001) groups had a lower risk of CVD death compared to the T1 group **(** Table 2).

**TABLE 2:** Associations between ALI and cardiovascular mortality in NHANES 1999–2018 followed through 2019.

Variable	Model 1			Model 2			Model 3		
	HR	95% CI	*p*-value	HR	95% CI	*p*-value	HR	95% CI	*p*-value
Continuous variables									
ALI per 10 U	0.84	0.81–0.87	<0.001	0.92	0.90–0.94	<0.001	0.89	0.87–0.92	<0.001
Tripartite variable									
T1 group		Ref			Ref			Ref	
T2 group	0.47	0.41–0.53	<0.001	0.64	0.56–0.72	<0.001	0.59	0.50–0.69	<0.001
T3 group	0.32	0.27–0.38	<0.001	0.56	0.48–0.65	<0.001	0.48	0.39–0.58	<0.001

Model 1: No adjusted.

Model 2: Adjusted by age, gender.

Model 3: Adjusted by age, gender, race/ethnicity, smoke, drink, BMI, cr, TG, TC, glu, CHF, CHD, DM, stroke, antihypertensive drugs, cancer, HEI-2015, DBP, SBP.

### Restricted regression cubic spline

**FIGURE 3:**
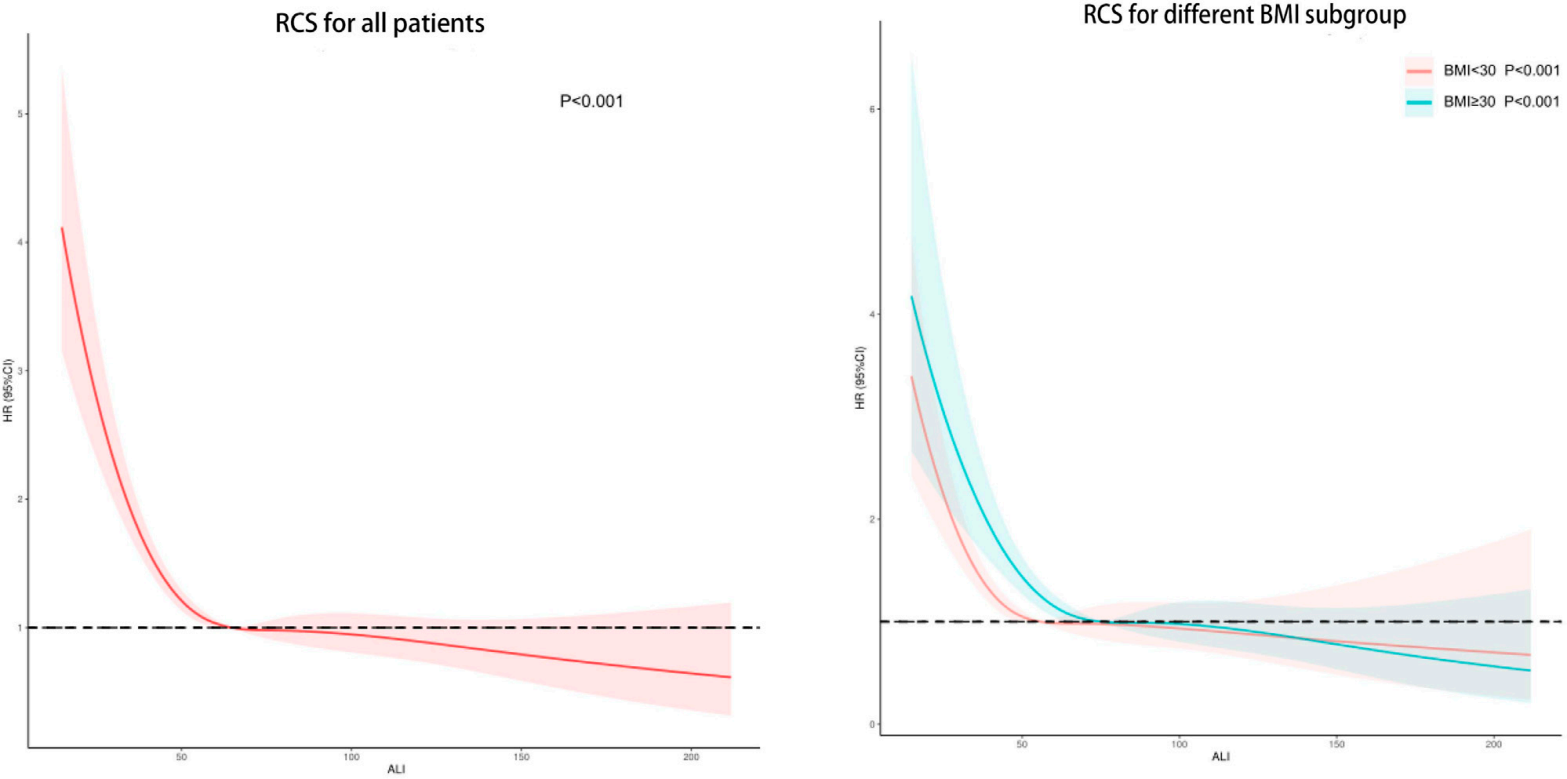
Potential nonlinear relationship between ALI and cardiovascular death in hypertensive patients (weighted).

The results of the restricted RCS analysis indicated no non-linear relationship between ALI and CVD death in hypertensive patients, and low levels of ALI were associated with an increased risk of CVD death in this population. Stratification by BMI did not significantly alter the results. (Figure 3).

### Subgroup analysis

**FIGURE 4:**
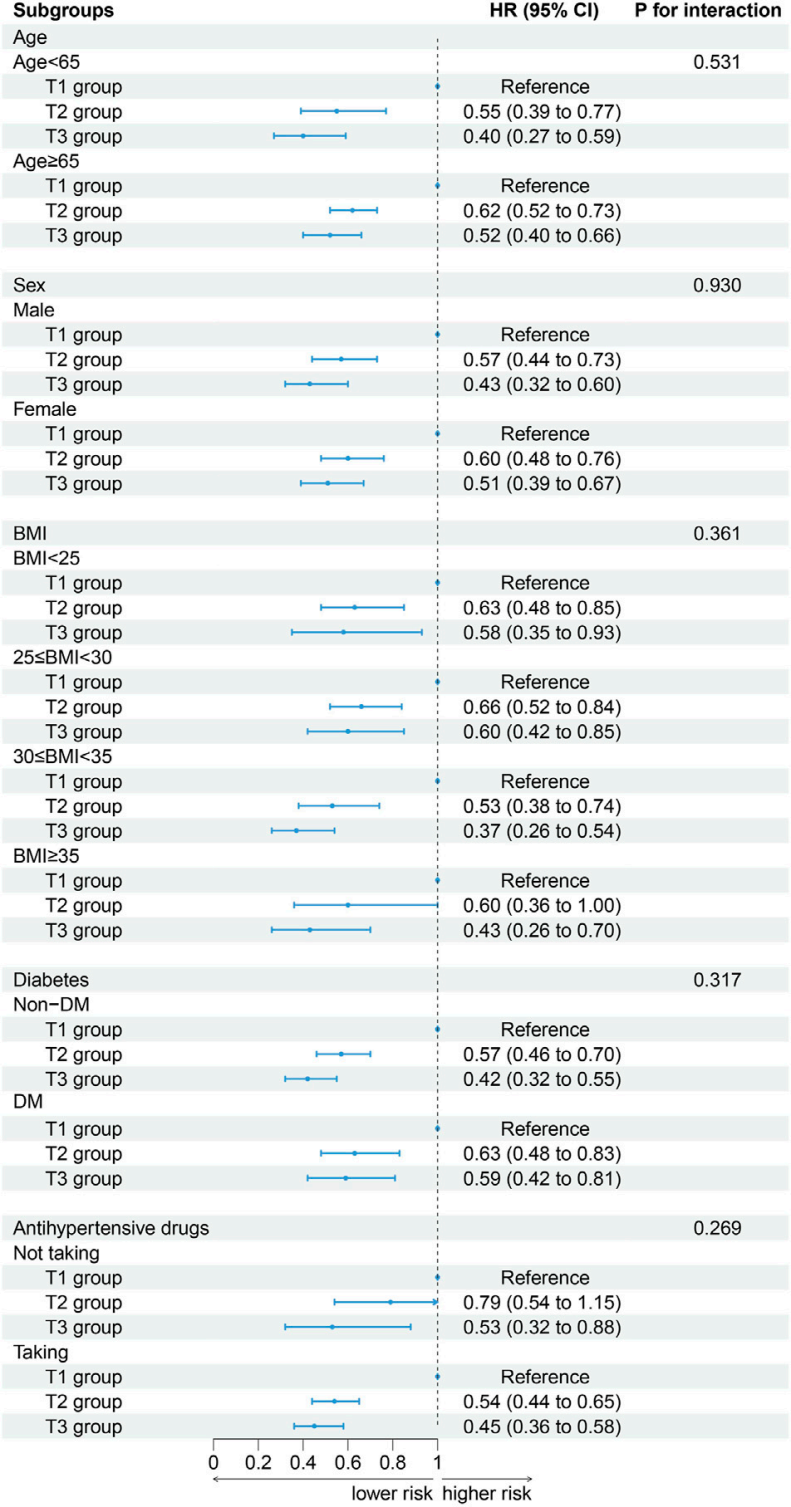
Association between ALI and cardiovascular mortality by selected subgroups (weighted).

When participants were stratified by age (P for interaction = 0.531), sex (P for interaction = 0.930), BMI (P for interaction = 0.361), DM (P for interaction = 0.317) and antihypertensive drugs (P for interaction = 0.269) the association between ALI and CVD mortality did not change. With the increased of ALI, the risk of CVD death decreased. The results of a stratified analysis by drug were consistent with the main effect (Figure 4).

#### Supplementary analysis

Among the items required for ALI calculations, alb and NLR were associated with the risk of CVD death in hypertensive patients after full adjustment for confounding variables (Supplementary Table S1). After grouping by BMI quintile, groups Q2, Q3, and Q4 had a lower risk of CVD death compared to group Q1. The Q5 group was not statistically significant (Supplementary Table S2). In addition, the results of a subgroup analysis of ALI showed that elevated ALI was associated with a reduced risk of CVD death in hypertensive patients in the subgroup with ALI≤60. *p* values were not statistically significant in the subgroup with ALI>60, despite a downward trend in CVD death risk (Supplementary Table S3).

## Discussion

This cohort study conducted in the United States utilized ALI to evaluate the inflammatory status of hypertensive patients. The findings indicated that patients with higher ALI had a decreased risk of CVD death, even after controlling for various confounding factors. This association remained consistent across different age groups, genders, BMI categories, and DM status. These results suggest that lower levels of inflammation were associated with a lower risk of CVD death in individuals with hypertension.

Hypertension was considered to be disease associated with inflammation (Steven et al., 2019). The chronic, low-grade inflammatory state in hypertensive patients can contribute to vascular remodeling, leading to vascular fibrosis and an increased risk of CVD-related mortality. (Intengan and Schiffrin, 2001). In a prospective study, hypertension patients with higher levels of inflammation had a 2-fold increased risk of all-cause death and a 1.8-fold increased risk of CVD (Cortez et al., 2016). The findings of these studies all suggest that inflammation was detrimental prognostic factors in patients with hypertension, which was in line with our results. However, it was worth noting that prior research has typically relied on a single inflammatory marker to evaluate the prognosis of hypertensive patients (Engström et al., 2006; Cortez et al., 2016; Sun et al., 2017). As mentioned in the introduction, inflammation can accelerate protein breakdown, resulting in a reduction in albumin levels. Moreover, inflammation can also induce insulin resistance, diminish appetite, and hinder the absorption of nutrients, ultimately leading to weight loss (Yoon et al., 2016; Guescini et al., 2017; Merker et al., 2020; Aldhwayan et al., 2022; Dou et al., 2022). Hence, relying solely on a single inflammatory marker to evaluate the mortality risk in patients with hypertension may not provide sufficiently accurate results.

ALI was calculated as BMI (kg/m^2^) × serum albumin level (g/dL)/NLR. Due to the fact that inflammation often results in hypoproteinemia and decreased BMI, previous studies have combined these two parameters with inflammatory markers to comprehensively evaluate the systemic levels of inflammation in cancer patients. It has been reported that lung cancer patients with higher ALI have a reduced risk of death (Jafri et al., 2013). In non-cancer populations, the effectiveness of ALI has also been demonstrated. Several studies have shown that low ALI is associated with increased risk of coronary artery calcification, readmissions and death in patients with heart failure (Maeda et al., 2020; Fan et al., 2021; Yuan et al., 2022). However, few studies have utilized ALI to evaluate the risk of CVD death in patients with hypertension. Our findings demonstrate that higher ALI levels are associated with a reduced risk of CVD death in patients with hypertension. Moreover, the results remained consistent when stratified by age, sex, BMI, antihypertensive drug use, and DM.

As high BMI is a well-established risk factor for CVD mortality, it is crucial to account for this factor when assessing the link between ALI levels and CVD mortality in hypertensive patients (Li et al., 2020). Therefore, we adjusted for BMI and utilized RCS to examine the possible non-linear relationship between ALI and hypertension. After controlling for BMI, we found that higher levels of ALI remained associated with a lower risk of CVD mortality in hypertensive patients. While elevated ALI levels may correspond to higher BMI levels, our RCS analysis did not reveal any U-shaped relationship. Although the regression analysis indicated that an elevated BMI was linked to a higher risk of CVD mortality when treated as a continuous variable, this association was not significant (*p* = 0.091). On the other hand, our findings revealed that hypertensive patients with a BMI ranging from 24.9 to 35.5 kg/㎡ had a lower risk of CVD mortality compared to those with a BMI ≤24.9 kg/㎡. Consequently, the results of the regression analysis of BMI as a continuous variable may have been influenced by the severely obese population (BMI >35.5 kg/㎡), leading to inconsistent findings. Similar results have been reported in previous studies. For instance, a retrospective study showed that low BMI was linked to an increased 3-year risk of all-cause mortality in hypertensive patients, while obesity was related to a reduced risk of all-cause mortality (Kim et al., 2022). In Zhu et al. (2022) ‘s study, being underweight was associated with higher mortality in people with high blood pressure, while being overweight was associated with lower mortality. In addition, the study of Zhou et al. (2021) also confirmed that low BMI was an independent risk factor for death in patients with hypertension, while high BMI was not Obese individuals may have greater metabolic reserves to cope with inflammation and metabolic demands (Doehner et al., 2010). In addition, systemic vascular resistance and plasma renin activity were lower in hypertensive patients with higher BMI compared with those with lower BMI, which can improve the prognosis of hypertensive patients (Lavie et al., 2007). Therefore, although high BMI is a recognized risk factor for CVD death, ALI can still be used as a biomarker for prognosis in hypertensive patients with BMI≤35.5kg/㎡.

In the supplementary analysis, it was found that increased levels of ALI were associated with a decreased risk of CVD mortality only in the subgroup with ALI ≤60, which is in line with the RCS findings. This could be attributed to the fact that high ALI may be linked to high BMI, as previously mentioned. The accuracy of ALI may be significantly impacted when BMI >35.5 kg/㎡, indicating that ALI might not be suitable for individuals with severe obesity.

Previous studies have suggested that low ALI is associated with a higher risk of death and may require early intervention (Jafri et al., 2013; Maeda et al., 2020). increasing the intake of nutraceuticals and fruits has been shown to be associated with lower levels of inflammation (Scicchitano et al., 2014; Maaliki et al., 2019; Zuraini et al., 2021). As this was a retrospective cohort study, we were unable to confirm whether the use of nutritional supplements and increased fruit intake could improve the risk of CVD death in hypertensive patients. Therefore, these are only hypotheses for the treatment and management of hypertension, and more clinical trials are needed to confirm their effectiveness.

This cohort study, which included 20,517 individuals, has a large sample size that lends reliability to our findings. Our results indicate that hypertensive patients with high ALI levels have a lower risk of CVD death. As a simple and easily calculated index, ALI may provide a more comprehensive assessment of the risk of CVD death in hypertensive patients than a single inflammatory parameter. Early intervention in hypertensive patients with low ALI levels may potentially have a positive effect in reducing the risk of CVD death. However, further experimental studies are needed to confirm this hypothesis.

## Limitations

There are some limitations in our study. First, the diagnosis of hypertension and comorbidities was mostly based on self-reported information, which may have introduced recall bias. Second, the use of a single blood draw may not have provided a complete picture of a patient’s physical state, which could change over long-term follow-up. Finally, our study design as a cohort study means that the results should be interpreted as correlational rather than causal, given the possibility of unmeasured confounding factors (Reddy et al., 2018). Therefore, further clinical trials are necessary to confirm our findings.

## Conclusion

ALI was an effective indicator of systemic inflammation in hypertensive patients. High levels of ALI were associated with a reduced risk of CVD death in hypertensive patients.

## Data Availability

The raw data supporting the conclusion of this article will be made available by the authors, without undue reservation.
