# Supplementary material for: Advanced lung cancer inflammation index is associated with long-term cardiovascular death in hypertensive patients: national health and nutrition examination study, 1999–2018
Source: Front Physiol. 2023 May 3;14:1074672. doi: 10.3389/fphys.2023.1074672 (PMC10189044; doi:10.3389/fphys.2023.1074672)
Supplement: Supplementary file 1 [file Table1.docx]

**Supplementary Table 1. Associations between BMI, Albumin, NLR and cardiovascular mortality in NHANES 1999–2018 followed through 2019.**

| **Variable** |  | **Model 1** | | |  | **Model 2** | | |  | | **Model 3** | | |
| --- | --- | --- | --- | --- | --- | --- | --- | --- | --- | --- | --- | --- | --- |
|  |  | **HR** | **95% CI** | ***P*-value** |  | **HR** | **95% CI** | ***P*-value** |  | | **HR** | **95% CI** | ***P*-value** |
| **Continuous variables** | | |  |  |  |  |  |  | |  |  |  |  |
| BMI | | 0.98 | 0.97-0.99 | <0.001 |  | 1.02 | 1.01-1.03 | <0.001 | |  | 1.01 | 1.00-1.03 | 0.091 |
| Albumin | | 0.31 | 0.26-0.37 | <0.001 |  | 0.36 | 0.29-0.44 | <0.001 | |  | 0.41 | 0.32-0.53 | <0.001 |
| NLR | | 1.22 | 1.16-1.28 | <0.001 |  | 1.14 | 1.08-1.20 | <0.001 | |  | 1.10 | 1.04-1.16 | 0.001 |
| **Per SD** | |  |  |  |  |  |  |  | |  |  |  |  |
| BMI | | 0.55 | 0.39-0.77 | <0.001 |  | 2.21 | 1.51-3.24 | <0.001 | |  | 1.54 | 0.93-2.56 | 0.091 |
| Albumin | | 0.01 | 0.00-0.02 | <0.001 |  | 0.01 | 0.00-0.03 | <0.001 | |  | 0.02 | 0.01-0.06 | <0.001 |
| NLR | | 1.69 | 1.49-1.92 | <0.001 |  | 1.45 | 1.25-1.68 | <0.001 | |  | 1.28 | 1.10-1.49 | 0.001 |

Model 1: No adjusted.

Model 2: Adjusted by age, gender.

Model 3: Adjusted by age, gender, race/ethnicity, smoke, drink, BMI, Cr, TG, TC, Glu, CHF, CHD, DM, stroke, antihypertensive drugs, cancer, HEI-2015, DBP, SBP.
