# Supplementary figures and images for: Advanced lung cancer inflammation index is associated with long-term cardiovascular death in hypertensive patients: national health and nutrition examination study, 1999–2018
Source: Front Physiol. 2023 May 3;14:1074672. doi: 10.3389/fphys.2023.1074672 (PMC10189044; doi:10.3389/fphys.2023.1074672)

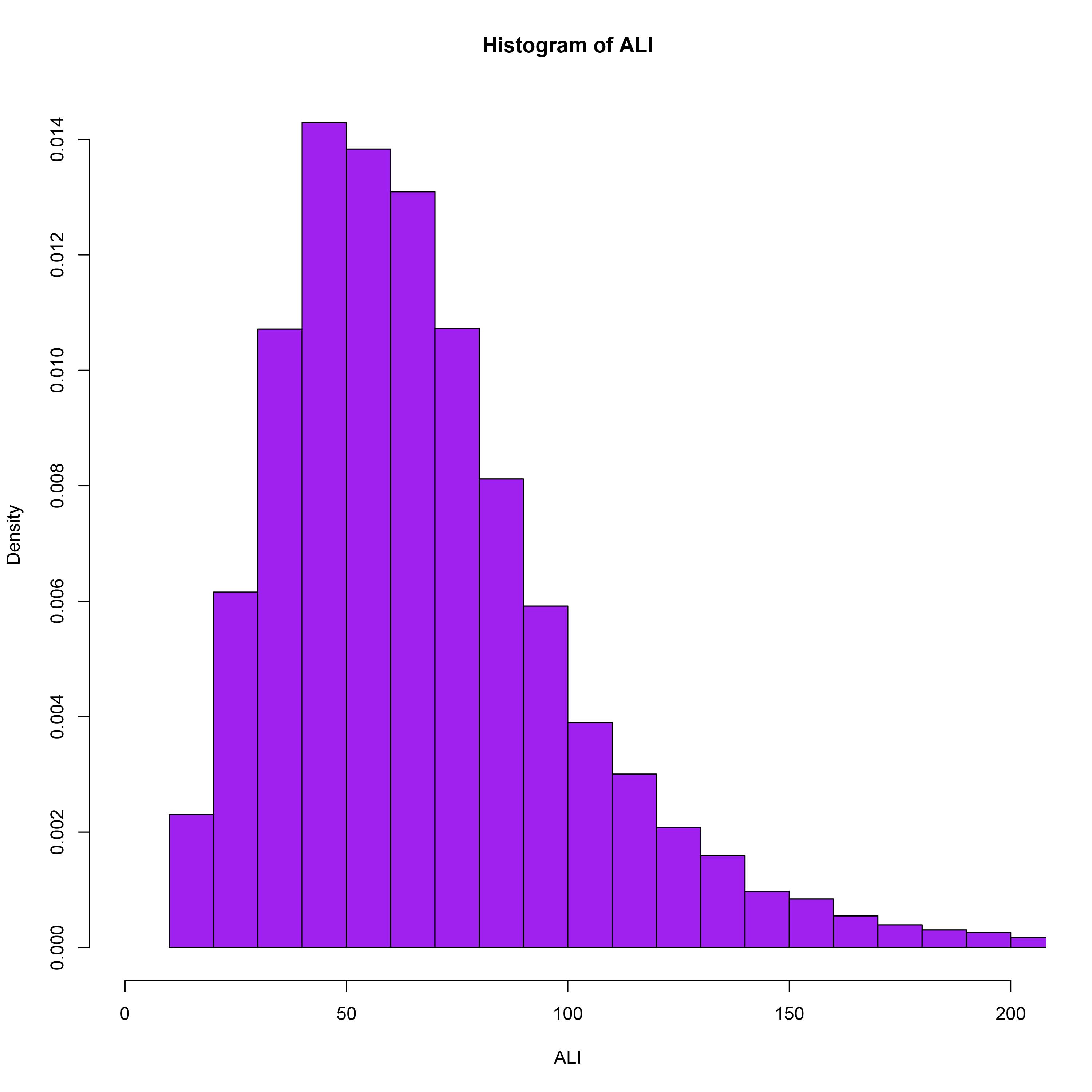

Supplement: Supplementary file 2 [file Image1.JPEG]
