# Supplementary material for: Advanced lung cancer inflammation index is associated with long-term cardiovascular death in hypertensive patients: national health and nutrition examination study, 1999–2018
Source: Front Physiol. 2023 May 3;14:1074672. doi: 10.3389/fphys.2023.1074672 (PMC10189044; doi:10.3389/fphys.2023.1074672)
Supplement: Supplementary file 3 [file Table2.docx]

**Supplementary Table 2. Associations between BMI (quintile) and cardiovascular mortality in NHANES 1999–2018 followed through 2019.**

| **Variable** |  | **Model 1** | | |  | **Model 2** | | |  | | **Model 3** | | |
| --- | --- | --- | --- | --- | --- | --- | --- | --- | --- | --- | --- | --- | --- |
|  |  | **HR** | **95% CI** | ***P*-value** |  | **HR** | **95% CI** | ***P*-value** |  | | **HR** | **95% CI** | ***P*-value** |
| Q1 | | Ref | | |  | Ref | | | |  | Ref | | |
| Q2 | | 0.73 | 0.61-0.88 | <0.001 |  | 0.73 | 0.61-0.86 | <0.001 | |  | 0.67 | 0.55-0.82 | <0.001 |
| Q3 | | 0.74 | 0.60-0.92 | 0.010 |  | 0.86 | 0.71-1.03 | 0.106 | |  | 0.74 | 0.60-0.92 | 0.006 |
| Q4 | | 0.60 | 0.51-0.72 | <0.001 |  | 0.83 | 0.70-0.99 | 0.039 | |  | 0.70 | 0.56-0.86 | <0.001 |
| Q5 | | 0.64 | 0.53-0.78 | <0.001 |  | 1.42 | 1.16-1.75 | <0.001 | |  | 1.05 | 0.80-1.37 | 0.741 |

Q1:BMI≤ 24.90 kg/㎡; Q2: 24.90 kg/㎡<BMI≤27.97 kg/㎡; Q3: 27.97 kg/㎡<BMI≤31.05 kg/㎡; Q4: 31.05 kg/㎡<BMI≤35.50 kg/㎡; Q5: BMI>35.50 kg/㎡

Model 1: No adjusted.

Model 2: Adjusted by age, gender.

Model 3: Adjusted by age, gender, race/ethnicity, smoke, drink, BMI, Cr, TG, TC, Glu, CHF, CHD, DM, stroke, antihypertensive drugs, cancer, HEI-2015, DBP, SBP.
