# Supplementary material for: Advanced lung cancer inflammation index is associated with long-term cardiovascular death in hypertensive patients: national health and nutrition examination study, 1999–2018
Source: Front Physiol. 2023 May 3;14:1074672. doi: 10.3389/fphys.2023.1074672 (PMC10189044; doi:10.3389/fphys.2023.1074672)
Supplement: Supplementary file 4 [file Table3.docx]

**Supplementary Table 3. Relationship between ALI and CVD death in hypertensive patients in different ALI subgroups**

| **Variable** |  | **Model 1** | | |  | **Model 2** | | |  | | **Model 3** | | |
| --- | --- | --- | --- | --- | --- | --- | --- | --- | --- | --- | --- | --- | --- |
|  |  | **HR** | **95% CI** | ***P*-value** |  | **HR** | **95% CI** | ***P*-value** |  | | **HR** | **95% CI** | ***P*-value** |
| **ALI≤60** | | |  |  |  |  |  |  | |  |  |  |  |
| ALI per 1 U | | 0.96 | 0.95-0.96 | <0.001 |  | 0.97 | 0.96-0.98 | P<0.001 | |  | 0.97 | 0.96-0.98 | <0.001 |
| ALI per 10 U | | 0.65 | 0.60-0.69 | <0.001 |  | 0.75 | 0.70-0.81 | <0.001 | |  | 0.73 | 0.68-0.79 | <0.001 |
| **ALI>60** | | |  |  |  |  |  |  | |  |  |  |  |
| ALI per 1 U | | 1.00 | 0.99-1.00 | 0.008 |  | 1.00 | 1.00-1.00 | 0.581 | |  | 1.00 | 0.99-1.00 | 0.164 |
| ALI per 10 U | | 0.95 | 0.92-0.99 | 0.008 |  | 0.99 | 0.96-1.02 | 0.581 | |  | 0.97 | 0.93-1.01 | 0.164 |

Model 1: No adjusted.

Model 2: Adjusted by age, gender.

Model 3: Adjusted by age, gender, race/ethnicity, smoke, drink, BMI, Cr, TG, TC, Glu, CHF, CHD, DM, stroke, antihypertensive drugs, cancer, HEI-2015, DBP, SBP.
